# The Growth and Scope of Voluntary Hospitals in London

**Published:** 1897-08-21

**Authors:** 


### The Institutional Workshop.

### THE GROWTH AND SCOPE OF VOLUNTARY HOSPITALS IN LONDON.

From a Correspondent.

### V.—THE FIRST DISPENSARIES.

In order to find the origin of this term applied to the medical relief of the poor, it is necessary to go as far back as the end of the seventeenth century. The condition of the medical profession at that period was peculiar. The Royal College of Physicians held nominally and legally a strict monopoly of the practice of physic in London and seven miles round. They had it in their power to restrain all unlicensed persons from practice, and they did "ever and anon make an example of one or other of them to strike terror into the rest." But it was in vain that they endeavoured to bind the people of London to trust themselves exclusively to the regular members of the profession. Whether it were that the public confidence had been too rudely shaken by the impotence and cowardice of the doctors in the plague season, still in the memories of many, whether the fees demanded were as oppressive as writers of the period imply, or whether the examinations were purely perfunctory, and the licence represented no real qualification—for whatever reason it is perfectly clear that doctors were held at this time in the lowest esteem. They complained loudly and bitterly of want of practice, and were so far from perceiving their own deficiencies, that they laid all the blame at the door of the "Chymists and Distillers, Astrologers, and Mountebanks, Midwives, and the whole train of broken-Tradesmen-Doctors," who ousted them from their patients. But "hardly all of them (they continue) have invaded the tenth part of the practice that one single sort of men have usurped, viz., the apothecaries. And these not only practice, but pretend to a right and a necessity so to do." The growing fervour of discontent resolved itself, in fact, into a quarrel *à outrance* with the College of Apothecaries.

The apothecaries undoubtedly held at this time a very strong position. Drug-taking had become a passion with a vast number of people, and there seems to be little doubt that the craze had been fostered by dishonest physicians, who played into the hands of the apothecaries. Report said that commissions of 25 and even 50 per cent. were paid on all drugs to the physician introducing the patient, and it seems clear that, at any rate, some sort of unholy alliance often subsisted between dispenser and prescriber. The profits made on drugs were the more enormous from the mysterious character of many of the ingredients prescribed. Mithridatum and the varieties of treacle, for instance, had a purely fancy value, and from the number of their component parts lent themselves with peculiar readiness to adulteration and shams. The Bezoar stone, again, a secretion found in the bodies of certain goats in the East, was greatly in request for almost every malady. Very little of the real stone was imported, and this at a high price, as much as 2,000 livres being given for a stone weighing four ounces. A spurious Bezoar stone was, however, discovered in the stomachs of German cows, and this cheap commodity, "made in Germany," was put into almost every prescription, and made the excuse for exorbitant charges. Pearl powder and so-called essence of gold figure freely also in the prescriptions of the period, and the bills were further swollen by the practice of splitting up a half-crown bottle of mixture (worth perhaps 2d. or 3d.) into eight or ten portions of 1s. each. The drug bill for a wealthy family would amount to £300 every Christmas. The bill of a lady "destroyed by vapours and medicines" is quoted at £175 15s. 6d. Three or four guineas a day was the usual cost of medicines for a wealthy person in illness, and in the course of a short fever he would consume from thirty to fifty pounds worth of drugs. Those in poorer circumstances might think themselves lucky if they escaped at a guinea a day. The apothecaries were ever ready to add free attendance to their drugs, and thus gained an entrance into many houses where doctors' bills were dreaded. More especially they plumed themselves on giving free attendance to the poor, and a sense of the ground thus lost to their adversaries roused at length the College of Physicians to action.

In 1697 it was voted that all members of the College, whether Fellows, Candidates, or Licentiates, should give their advice gratis to all their sick neighbouring poor, when desired, within the City of London and seven miles round. They communicated this vote to the Lord Mayor and Common Council, who returned thanks to them for their charitable order, but with characteristic civic caution desired "that they would explain themselves as to whom they meant by poor." This intricate question, which the wits of two centuries has not yet succeeded in settling, was answered by the College in a manner worthy of attention. The onus of proving their own poverty was put on the patients themselves, and it was decreed that all those should be esteemed poor that brought certificates under the hand of the rector, vicar, or curate of the parish in which they lived, of their being such. The physicians were now free from the imputation so often cast against them of giving no attendance without a fee, but it does not appear that their services were at first very eagerly sought after. It was found that the high price of the commonest drugs rendered their prescriptions practically useless to the poorest class. Accordingly, the following year the laboratory of the College was fitted up for preparing medicines for the poor, and the apothecaries were formally invited to co-operate by contributing drugs at cost price. The long smouldering rivalry at once burst into a blaze. In the contest which ensued the College of Physicians seems to have had all the talent and most of the honesty on their side. The apothecaries refused to aid in establishing a fixed low rate at which the poor might procure their medicines, and threatened with the heaviest penalties certain "honest and charitable apothecaries" who undertook to make up the prescriptions of the physicians for little over cost price. Thus threatened with the failure of their whole scheme, some forty of the leading physicians of the College, among whom the name of Hans Sloane is conspicuous, furnished a subscription of ten guineas apiece, and in the teeth of the fiercest opposition from many of their own profession, proceeded to found a dispensary in Warwick Lane, in the neighbourhood of the College. Joint committees were formed of members of the College of Physicians, the Court of Aldermen and Common Council to manage the undertaking; and, at the motion of the City members, the term "poor" was held to include "all hired servants and apprentices to handicraft men." Two physicians gave attendance gratis on certain days, and medicines were sold at the dispensary on a doctor's order at their intrinsic value. It was found necessary to advertise this charitable plan in the public prints and in papers distributed among the poor; and so successful was the undertaking that in 1702 two more dispensaries, one in St. Martin's Lane, and the other in Gracechurch Street, were established on the same principles. Several apothecaries in different parts of the City consented to brave the wrath of their colleagues, and make up prescriptions for the poor at the price which the physicians wrote in at the bottom. The benefits of these low prices were restricted to "poor housekeepers and their families that did not receive alms," but the churchwardens and overseers were not long before they obtained permission to buy drugs for parochial use on the same terms. As time went on the physicians who gave their services in this manner, and were commonly known as dispensarians, began to be accused of sending their private patients to buy their drugs at the new dispensaries. The incapacity of the apothecaries, many of whom bought even pills and draughts ready concocted from wholesale dealers, the common practice of storing three qualities of drugs and using chiefly the inferior, or substituting one for another, with easy disregard for consequences, lent some justification to this infringement of trade rights, and it is easy to understand that patients made no objection to a system which saved their pockets 50 or 75 per cent. on the cost of each article. But it is also clear that the "out-patient abuse" had already begun, and that the physicians were liable to be deceived by some who came in *forma pauperis* to get free advice. A heavy blow was struck at the extortions and malpractices of the apothecaries.

It will be seen, then, that as early as the beginning of the eighteenth century, three dispensaries were in full working order, with aims not dissimilar to those of modern days, offering gratuitous medical advice and medicine at cost price. It was the proud boast of the dispensarians "that the poor have that care taken of them and kindness showed them that we challenge any city in Europe to parallel."

